# Kidney-specific methylation patterns correlate with kidney function and are lost upon kidney disease progression

**DOI:** 10.1186/s13148-024-01642-w

**Published:** 2024-02-12

**Authors:** Naor Sagy, Noa Meyrom, Pazit Beckerman, Oren Pleniceanu, Daniel Z. Bar

**Affiliations:** 1 Department of Oral Biology, Goldschleger School of Dental Medicine, The Faculty of Medical and Health Sciences, Tel Aviv University, 69978 Tel Aviv, Israel; 2 Kidney Research Lab, The Institute of Nephrology and Hypertension, Sheba Medical Center, Tel-Hashomer and The Faculty of Medical and Health Sciences, Tel-Aviv University, Tel Aviv, Israel; 3 The AI and Data Science Center (TAD), Tel Aviv University, 69978 Tel Aviv, Israel

## Abstract

**Background:**

Chronological and biological age correlate with DNA methylation levels at specific sites in the genome. Linear combinations of multiple methylation sites, termed epigenetic clocks, can inform us the chronological age and predict multiple health-related outcomes. However, why some sites correlating with lifespan, healthspan, or specific medical conditions remain poorly understood. Kidney fibrosis is the common pathway for chronic kidney disease, which affects 10% of European and US populations.

**Results:**

Here we identify epigenetic clocks and methylation sites that correlate with kidney function. Moreover, we identify methylation sites that have a unique methylation signature in the kidney. Methylation levels in majority of these sites correlate with kidney state and function. When kidney function deteriorates, all of these sites regress toward the common methylation pattern observed in other tissues. Interestingly, while the majority of sites are less methylated in the kidney and become more methylated with loss of function, a fraction of the sites are highly methylated in the kidney and become less methylated when kidney function declines. These methylation sites are enriched for specific transcription-factor binding sites. In a large subset of sites, changes in methylation patterns are accompanied by changes in gene expression in kidneys of chronic kidney disease patients.

**Conclusions:**

These results support the information theory of aging, and the hypothesis that the unique tissue identity, as captured by methylation patterns, is lost as tissue function declines. However, this information loss is not random, but guided toward a baseline that is dependent on the genomic loci.

**Significance statement:**

DNA methylation at specific sites accurately reflects chronological and biological age. We identify sites that have a unique methylation pattern in the kidney. Methylation levels in the majority of these sites correlate with kidney state and function. Moreover, when kidney function deteriorates, all of these sites regress toward the common methylation pattern observed in other tissues. Thus, the unique methylation signature of the kidney is degraded, and epigenetic information is lost, when kidney disease progresses. These methylation sites are enriched for specific and methylation-sensitive transcription-factor binding sites, and associated genes show disease-dependent changes in expression. These results support the information theory of aging, and the hypothesis that the unique tissue identity, as captured by methylation patterns, is lost as tissue function declines.

**Supplementary Information:**

The online version contains supplementary material available at 10.1186/s13148-024-01642-w.

## Introduction

### Chronic kidney disease (CKD)

The human kidney plays several essential roles, including the excretion of toxic waste products and maintenance of blood pressure and pH. The average kidney contains ~ 1 million nephrons, which are its main functional units [1]. CKD is defined as loss of renal function, marked by decreased glomerular filtration rate (GFR) < 60 mL/min/1.73m^2^, and increase in kidney damage markers (e.g., albuminuria), or both, for ≥ 3 months [2]. Renal function can be assessed using serum creatinine (sCr) levels, used to compute the estimated Glomerular Filtration Rate (eGFR) via one of several formulae [3]. Unfortunately, sCr-based assessments are highly inaccurate for several reasons: (1) sCr is highly insensitive for diagnosing early CKD, as abnormalities in its values occur only when ~ 40% of the renal parenchyma is damaged [4]. Indeed, many individuals with normal sCr actually have kidney damage upon histological examination including interstitial fibrosis (IF), tubular atrophy (TA) and glomerulosclerosis (GS)—all indicative of impaired renal function [5–7]. CKD is a relentless disorder, steadily deteriorating from its early stages (1–2) in which the GFR is normal or high to stage 5, or end-stage kidney disease (ESKD), defined as a GFR < 15 ml/min/1.73m2, when patients require kidney replacement therapy (KRT): either dialysis or transplantation [2]. With a prevalence of up to 17.3% [8–10] requiring expensive treatments, CKD is a global epidemic, accounting for over 1.2 million deaths a year [11]. Moreover, its true prevalence is hard to determine, because its early stages often go undetected. CKD rates are expected to keep rising due to the aging of the population and growing prevalence of its 2 main risk factors: diabetes mellitus (DM) and hypertension (HTN) [11]. The irreversible nature and lack of specific treatments for CKD underscore the importance of its early diagnosis, while renal function is preserved and active interventions (e.g., glycemic control in DM) may slow its progression, which will improve quality of life, reduce mortality, and reduce the costs for healthcare systems [12].

## DNA methylation correlates with lifespan, healthspan and CKD

DNA methylation is an epigenetic modification, and the most studied one in relation to diseases, healthspan and lifespan. Seminal works by Horvath and Hannum described "epigenetic clocks," a linear combination of the methylation levels of several tens to hundreds of genomic sites, which accurately estimate the biological age of most human tissues, including the kidney [13–15]. These clocks have been expanded to predict “biological age,” capture multiple aspects of healthspan and predict all-cause mortality [13]. Similarly, methylation sites can be used as surrogates for plasma protein levels. Proving the relevance of epigenetic signatures to renal function, the Susztak group profiled genome-wide methylation of tubular cells in CKD and normal kidneys and found that a 65-probe signature correlates with kidney structural damage, as seen in biopsy, and a 471-probe signature predicts renal functional decline together with clinical parameters [16]. Likewise, it has been recently shown that the progression of CKD is tightly linked to methylation-induced changes in tubular cell function, including senescence [17]. Late stage CKD is also associated with increased biological age, as predicted by DNA methylation patterns in blood samples. This increase is reduced upon kidney transplant, but not by dialysis [18]. Remarkably, renal methylation signatures also accurately reflect the presence of kidney cancer, with the methylome of RCC cells exhibiting a significantly older biological age, a phenomenon seen in a wide range of cancers. Moreover, it has been shown that specific DNA methylation signatures predict various clinical outcomes and correlate with renal function [13, 19–21]. Although previous studies looked into the relation between DNA methylation and kidney diseases [16, 21–24], their selection of disease-associated methylation sites was extracted from comparing methylation arrays for healthy and sick kidney tissues. However, no analysis was done by pre-selecting sites predicted and validated to be correlated with disease progression.

## Changes in DNA methylation alter transcription-factor binding

DNA methylation often correlates with transcription levels. Epigenome-wide association studies (EWAS) using kidney and blood samples demonstrated that methylation at specific CpG sites are associated with kidney disease [22, 23, 25–29]. Methylation and demethylation can have direct effects on expression levels in vivo [30, 31], thus suggesting that some of the correlations might have a causal component to them. Methylation signatures can alter binding for some transcription factors (TF) [32, 33], which can mechanistically explain some of this effect. Indeed, EWAS studies, conducted in blood and validated in the kidney, found correlations between CpG methylation level and kidney expression levels of genes correlated with kidney function [21, 23].

Here, we identify epigenetic clocks and CpG sites that correlate with the functional state of the kidney, as captured by IF and eGFR. A subset of these CpG sites, selected in an undirected fashion, show a kidney-specific methylation pattern when compared to other tissues. Moreover, the majority of sites showing a kidney-specific methylation pattern correlate with IF. Multiple genes associated with these sites are changed in CKD. Interestingly, all these sites lose their unique methylation pattern in CKD, while trending toward the common form.

## Results

### Specific epigenetic clocks correlate with eGFR and IF

**Fig. 1:**
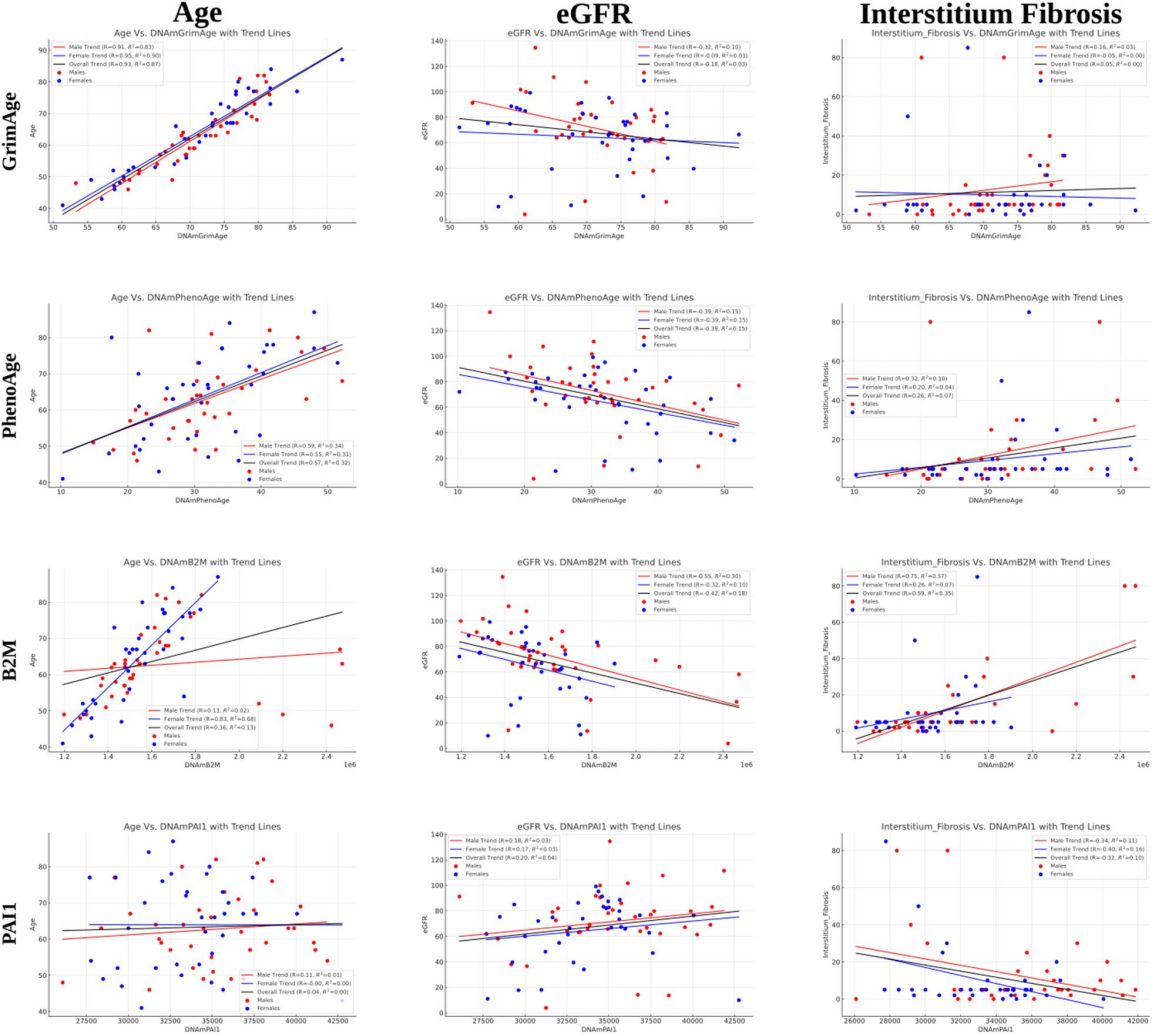
Methylation clocks correlate with kidney state in males. Correlation of age, eGFR and IF with four epigenetic clocks: GrimAge, PhenoAge, B2M and PAI1. Males—red; Females—blue. Regression line with all samples—black

CKD progression is accompanied by a decrease in eGFR and an increase in IF [5–7]. Several studies have analyzed the methylation of DNA in kidney tissue and identified CpGs whose methylation levels correlate with these markers of kidney disease progression [21, 22, 34]. To test whether eGFR decrease and IF increase are captured by existing epigenetic DNA methylation clocks, we analyzed 85 publicly available kidney methylation arrays [22, 34]. Samples were exclusively of kidney tissue, with an average age of 63 (standard deviation 11 years) and 53% males. Average eGFR was 67 (standard deviation 25). Multiple clocks showed a significant correlation with eGFR and IF (Fig. 1; Additional file 1: Table S1, S2). We noticed substantial differences in specific clock performance between males and females, thus we performed male and female analyses independently (Fig. 1; Additional file 1: Tables S2–S4). Of all clocks tested, Epigenetic Age (Zhang) and DNAmB2M, a surrogate for Beta-2-Microglobulin (B2M), performed best in males, giving correlations of − 0.60 and − 0.55 (*p* = 4 × 10^–4^) to eGFR and 0.75 (*p* = 7 × 10^–8^) to IF. By contrast, in females, DNAmPhenoAge gave a correlation of − 0.39 (*p* = 0.017) to eGFR and DNAmPAI1, a surrogate for Plasminogen Activator Inhibitor-1 (PAI1), a correlation of − 0.39 (*p* = 0.015) to IF. Age and BMI, two potential confounders, were poorly correlated with these clocks (Additional file 1: Tables S2–S4). In males, the age-adjusted B2M surrogate (DNAmB2MAdjAge) has a lower, but still significant correlation to both eGFR and IF. Similarly, results were obtained for PAI1 and IF in females. By contrast, age-adjusted PhenoAge in females did not significantly correlate with neither eGFR nor IF (Additional file 1: Tables S2–S4). We note that none of the tested clocks and surrogates were not designed to estimate kidney state, nor trained on relevant tissues. We concluded that some aspects of the physiological state of the kidney are reflected in the methylome and captured by epigenetic clocks not specifically developed for this purpose.

## Multiple CpG sites show strong correlation to eGFR and IF

**Fig. 2:**
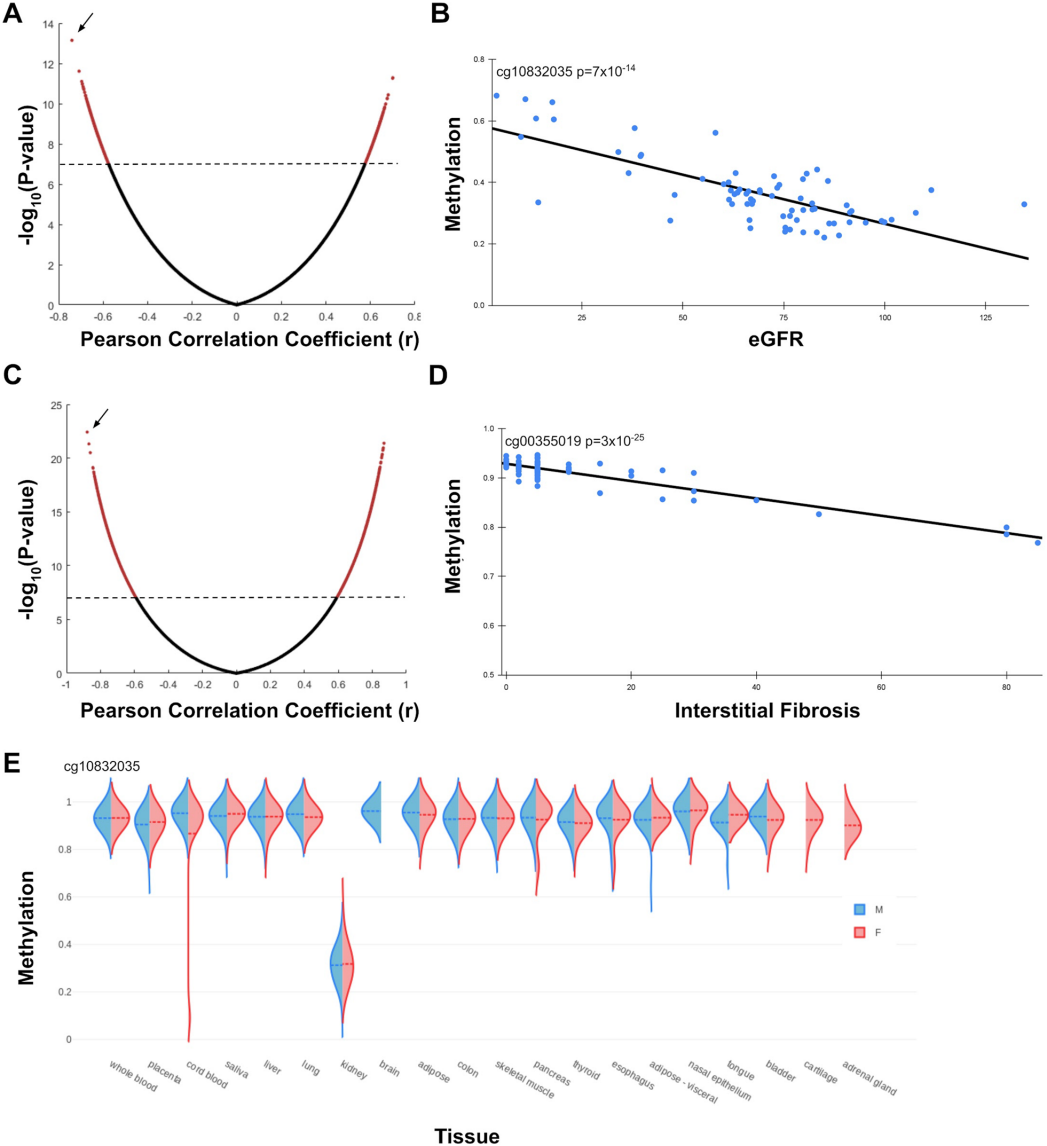
Individual CpG sites correlate with kidney state. **A.** Volcano plot of all tested CpG sites and their correlation to eGFR. Dots above the dashed line (red) pass Bonferroni correction. **B**. Individual methylation and eGFR levels for cg10832035, the site with highest correlation to eGFR (arrow in A). **C**. Volcano plot of all tested CpG sites and their correlation to IF.** D**. Individual methylation and eGFR levels for cg00355019, the site with highest correlation to IF (arrow in C). **E.** cg10832035 methylation patterns in a subset of tissues from the NGDC-CNCB dataset

Alternative methylation sites may provide an even better estimate of tissue state. Despite the small number of arrays, 1975 sites showed a statistically significant correlation (after Bonferroni correction) for eGFR, with correlation values up to *r *= 0.74 (Fig. 2A, B, Additional file 3: Figure S1; Table S5). We tested if these results can be explained by simple global loss or gain of methylation. Of the 1975 sites, 42% positively and 58% negatively correlated with eGFR, excluding global unidirectional changes as drivers of these methylation patterns. For IF, 20,986 sites passed Bonferroni correction, with r as high as 0.89 (Fig. 2C, D, Additional file 3:Figure S1; Additional file 1: Table S6). Of these, 67% showed a positive correlation and 33% a negative correlation. To test for the possibility that age, sex, race or BMI act as confounders, we measured their correlation with eGFR and IF in this dataset. Additionally, we examined the correlation of top 10 CpG sites with age and BMI in this and the independent NGDC-CNCB [35] dataset. In all cases, correlation to age, sex and BMI was weak or negligible, excluding them as confounders (Additional file 3: Figure S1; Additional file 1: Table S7). We took a closer look at cg10832035, the site with the highest correlation to eGFR (r = − 0.74; *p* < 10^–13^). As expected, it also showed a strong correlation to IF (r = 0.77, *p* < 10^–14^). cg10832035 was located on a CpG island inside the non-coding gene RP5-1086L22.1. The adjacent methylation sites within the island, but not outside the island, exhibited a similar methylation pattern and correlation to IF (r = 0.7; *p* < 10^–10^) and eGFR (r = − 0.64; *p* < 10^–8^), excluding possible probe issues. Interestingly, this site was almost completely methylated in all tissues except the kidney (Fig. 2E). In the kidney, methylation levels averaged around 0.35, and tended to increase with IF and decline in eGFR.

## CKD-correlated CpG sites are enriched for TF binding sites

**Fig. 3:**
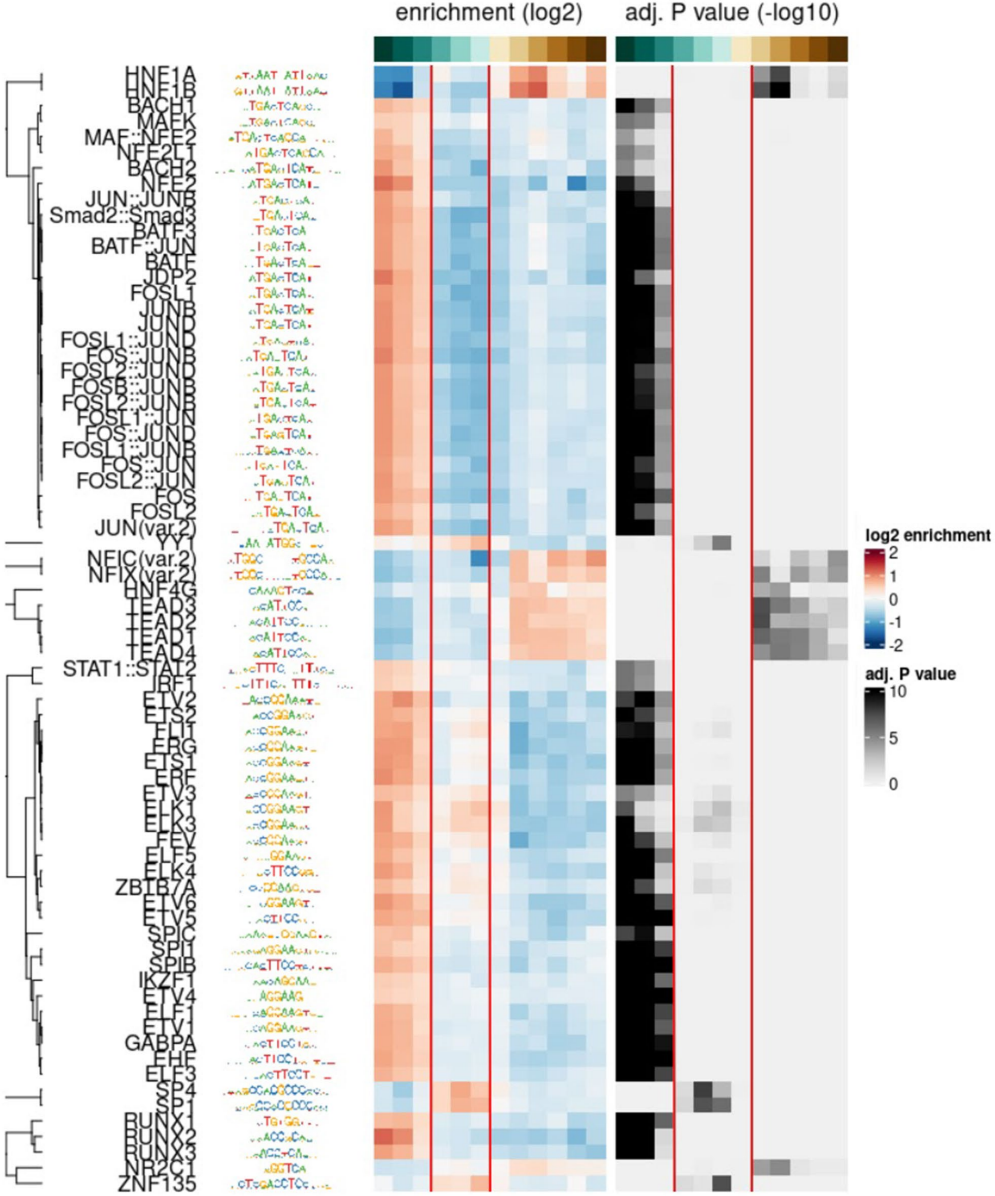
Methylation sites that correlate to IF are enriched for specific TF binding sites. TF enrichment and p-values for sequences around CpG sites that positively correlate with IF (left), do not correlate with IF (middle; between the red lines) and negatively correlate to IF (right)

To test for potential biological significance, sequences surrounding these CKD-correlated sites were used for motif enrichment analysis. Indeed, these sequences were enriched for TF binding sites. Specifically, sites that positively correlated with IF showed enrichment for the HNF1 family, while negatively correlated sites were enriched for Jun/FOS (Fig. 3). Gene associated with eGFR and IF correlated sites were used in functional annotation analysis (Additional file 1: Tables S8–S10; [36]), however associations were relatively weak.

## Kidney-specific methylation patterns are lost with kidney disease progression

Tissue-specific differentially methylated regions (tsDMRs) can have tissue-specific roles [37, 38]. After noticing the unique methylation pattern of cg10832035, we sought to test whether this is a general phenomenon. Using the NGDC-CNCB [35] dataset, we identified 427 methylation sites that exhibit a unique methylation pattern in the kidney (Additional file 1: Table S11). Of these sites, 68% were in the top 10% (*r* > 0.47; *p* < 4 × 10^–5^) of sites correlated with IF. Removing CpG sites with significant missing data increased this percentage to 72% (208 of 289; expected: 29; *p* = 10^–322^; Additional file 1: Table S11). Thus, the vast majority of uniquely methylated kidney sites exhibit changes in their methylation patterns as kidney function declines. To account for age bias, we analyzed the methylation distribution over a wide age range and observed no significant correlation between age and methylation for these kidney-unique sites (Additional file 3: Figure S2).

Two non-exclusive mechanisms can account for the observed changes to these kidney-specific methylation sites. Changes of cell populations and within cell populations may result in regression to the mean in these sites. Both mechanisms appear to contribute to this phenomenon [39]. Deconvolution analysis using EpiSCORE [40] found that the fraction of fibroblasts and immune cells, but not endothelial or epithelial cells, changed with IF (Additional file 3: Figure S3). These results may partially or fully account for the observed changes.

## Functional decline is accompanied by a drift toward the common methylation pattern

**Fig. 4:**
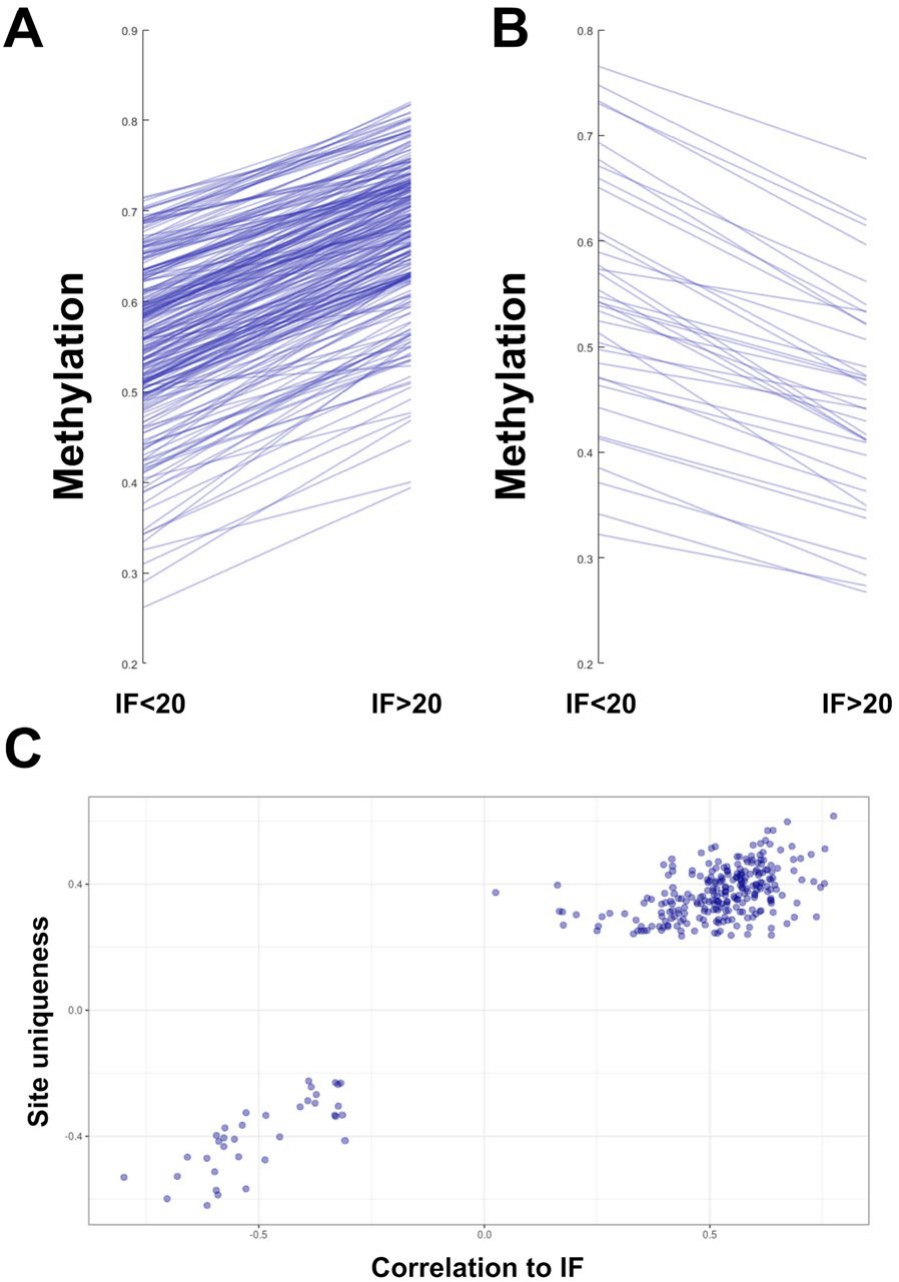
Methylation levels regress to the common baseline upon CKD. **A.** Methylation levels for the 253 sites undermethylated in the kidney, in individuals with IF < 20 and > 20. **B.** Methylation levels for the 36 sites overmethylated in the kidney, in individuals with IF < 20 and > 20. **C.** Site uniqueness, as measured by the distance from other tissues, and the corresponding correlation to IF

Cg10832035 is partially methylated in the kidney, but almost completely methylated in every other tissue examined (Fig. 3E). Indeed, we found this to be the common scenario. Of the 289 sites analyzed, 253 (88%) had a lower methylation level in the kidney as compared to the rest of the tissues, while only 36 (12%) had a higher methylation level. Upon kidney functional decline, cg10832035 becomes more methylated, trending toward the common methylation state of all other tissues examined. More generally, this raises three distinct possibilities, that upon functional decline (i) methylation levels increase; (ii) methylation levels trend toward the common form; (iii) methylation may increase or decrease independently of the common form. To distinguish between these three possibilities, we examined the levels of methylation in kidneys with different levels of fibrosis. For all 289 sites, methylation level trended toward the common methylation pattern found in the rest of the body (*p* = 10^–87^), increasing in the 253 sites that are less methylated in the kidney compared to other tissues (Fig. 4A) and decreasing in the 36 sites that are more methylated in the kidney (Fig. 4B). Moreover, there was a positive correlation between the site's “uniqueness,” quantified as its distance from methylation levels in all other tissues, and its correlation with the IF score (Fig. 4C; *r *= 0.95 *p* < 10^–148^). We conclude that the unique methylation pattern of the kidney erodes as kidney function declines.

## Characterization of kidney-specific methylation sites

Methylation patterns may be influenced by protein-DNA interaction, which in turn is guided by the primary DNA sequence [41]. To identify motifs that may help establish or maintain these unique methylation patterns, we extracted the sequences surrounding the 253 undermethylated and 36 overmethylated identified sites, and looked for motif enrichment. De novo motif discovery, using random CpG sites as background, identified multiple sequences showing strong enrichment (Additional file 3: Figure S4). Not surprisingly, these included significant enrichment for motifs identified in CpGs that correlated with CKD progression. To identify sequences that potentially contribute to the unique methylation pattern, while removing general CKD background, we repeated this analysis using as background CpG sites that correlate with IF, but do not have a unique methylation pattern. Again, multiple motifs were identified, including HNF1 and HIF2a (Additional file 3: Figure S5). However, the 253 sites identified differ significantly from both backgrounds. As most sites are almost fully methylated in all but one tissue, they may differ in GC content from the background. Indeed, the average GC percentage of both backgrounds, as well as the methylated-in-kidney sites, was ~ 55%. In contrast, the 253 sites, predominantly methylated in the body but not in the kidney, had an average GC content of 45%. Thus, we also compared these sites to those with unique methylation patterns in other tissues. Again, significant enrichment for specific transcription factors such as HNF1 was observed (Additional file 3: Figure S6).

Next, we analyzed methylation sites for tissue-specific differentially methylated positions (DMPs). eFORGE 2.0 [42] was used to identify such sites by overlap with DNase I hypersensitive sites (DHSs) compared to matched background. Only kidney-unique undermethylated sites had DHS (*p* < 10^–20^), and only in kidney (Additional file 3: Figure S7A, B). Next, we characterized the chromatin state of these sites. These sites were identified as enhancer sites (*p* < 10^–30^), but only in undermethylated sites and only in kidney (Additional file 3: Figure S7C, D).

## Expression change of genes associated with kidney-specific methylation patterns

**Fig. 5:**
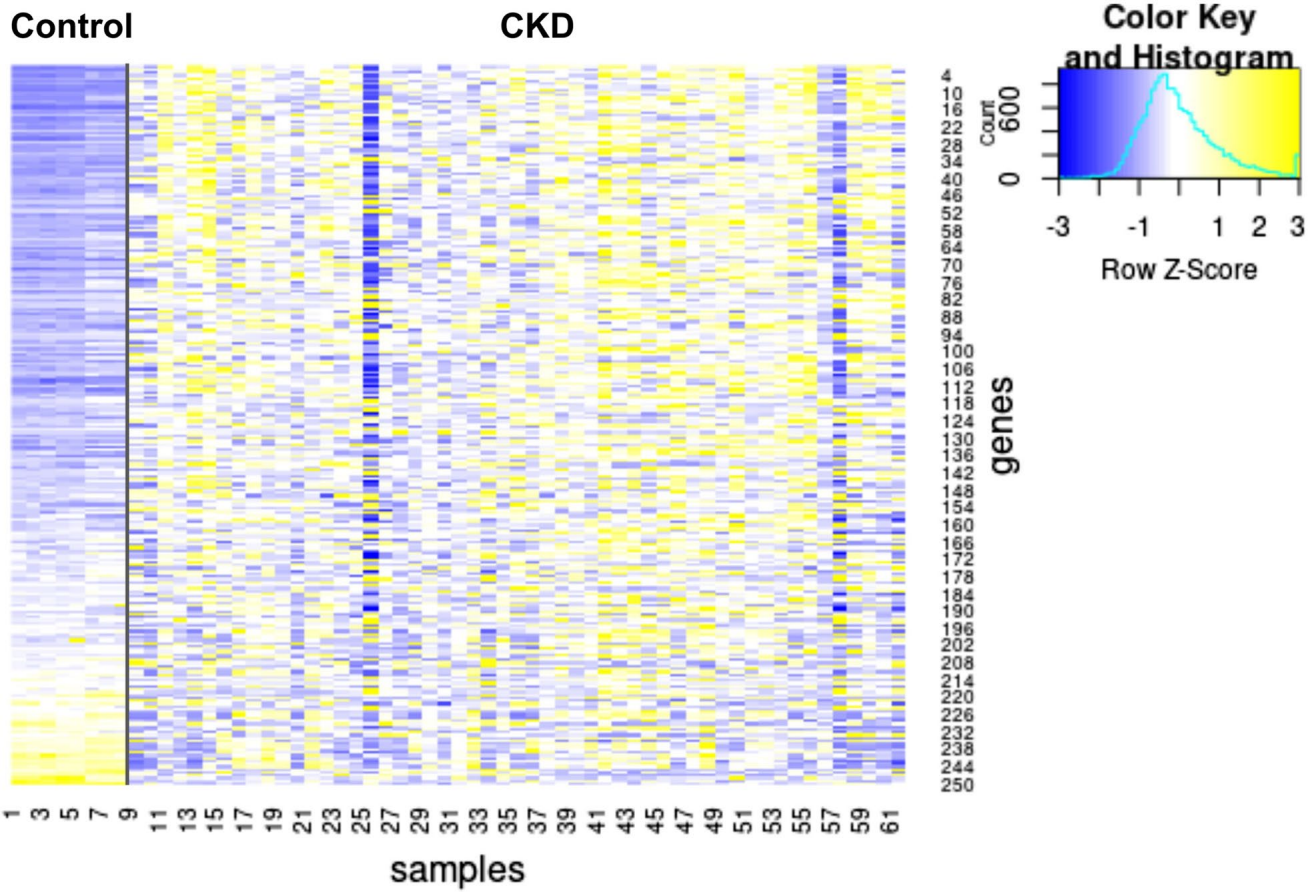
Genes associated with uniquely methylated sites change in expression in CKD. Expression levels of all (250) genes associated with uniquely methylated sites. Gene expression from the Nakagawa CKD Kidney dataset, with the discovery and validation cohorts united

Changes in methylation levels are often associated with changes in gene expression [41]. Depending on the genomic context, these changes can be causal or non-causal, and can involve an upregulation or downregulation of the associated gene [41, 43, 44]. We investigated whether alterations in kidney-specific methylation patterns correspond to changes in gene expression. To achieve this aim, we identified genes associated with the identified CpGs (Additional file 1: Table S11) and tested for changes in mRNA levels in kidney biopsies of CKD patients and controls [45]. Of the 250 genes tested, 210 (84%) showed a statistically significant (*p* < 0.05) difference between CKD and control (Additional file 1: Table S12). Of these, approximately 90% were upregulated in CKD patients (Fig. 5; Additional file 1: Table S12). We conclude that methylation at these specific CpG sites is correlated with gene expression changes, potentially reflecting alterations in local chromatin structure.

## Methylation sites negatively correlated with IF are linked to oncogenes and developmental genes

We clustered the 36 methylation sites negatively correlated with IF into 15 genomic regions which mapped to 13 genes (Additional file 1: Table S13). Of these 13 genes, seven are known as renal cancer prognostic genes (overexpression), five play a role in kidney differentiation and development, two of which overlap with cancer prognostic genes, and one functions as a tumor suppressor [46–52]. Kidney fibrosis is known to correlate with CKD and is subsequently with renal cancer [53, 54]. Levels of SPAG5, MCF2L, CCDC64, PDLIM4, EMX1, P4HA2, VIM and GRAMD1B methylation declined for IF > 20, which might indicate oncogenesis in the fibrotic tissue. Interestingly, CKD is a well-established risk factor for renal cancer [53, 54]. Although kidney inflammation [55] and relative immunodeficiency [56] have been suggested to play a role in CKD-related carcinogenesis, the exact molecular link has yet to be definitively established. Notably, although CKD results in a generalized inflammatory state [57], the risk of cancer in CKD has been shown to be limited to the kidney and not other organs [55], implying that it likely arises due to kidney-specific changes. The described methylation changes, which are highly kidney-specific, may thus account for at least some of the enhanced cancer risk among CKD patients.

## Epigenetic information loss correlates with IF and eGFR

**Fig. 6:**
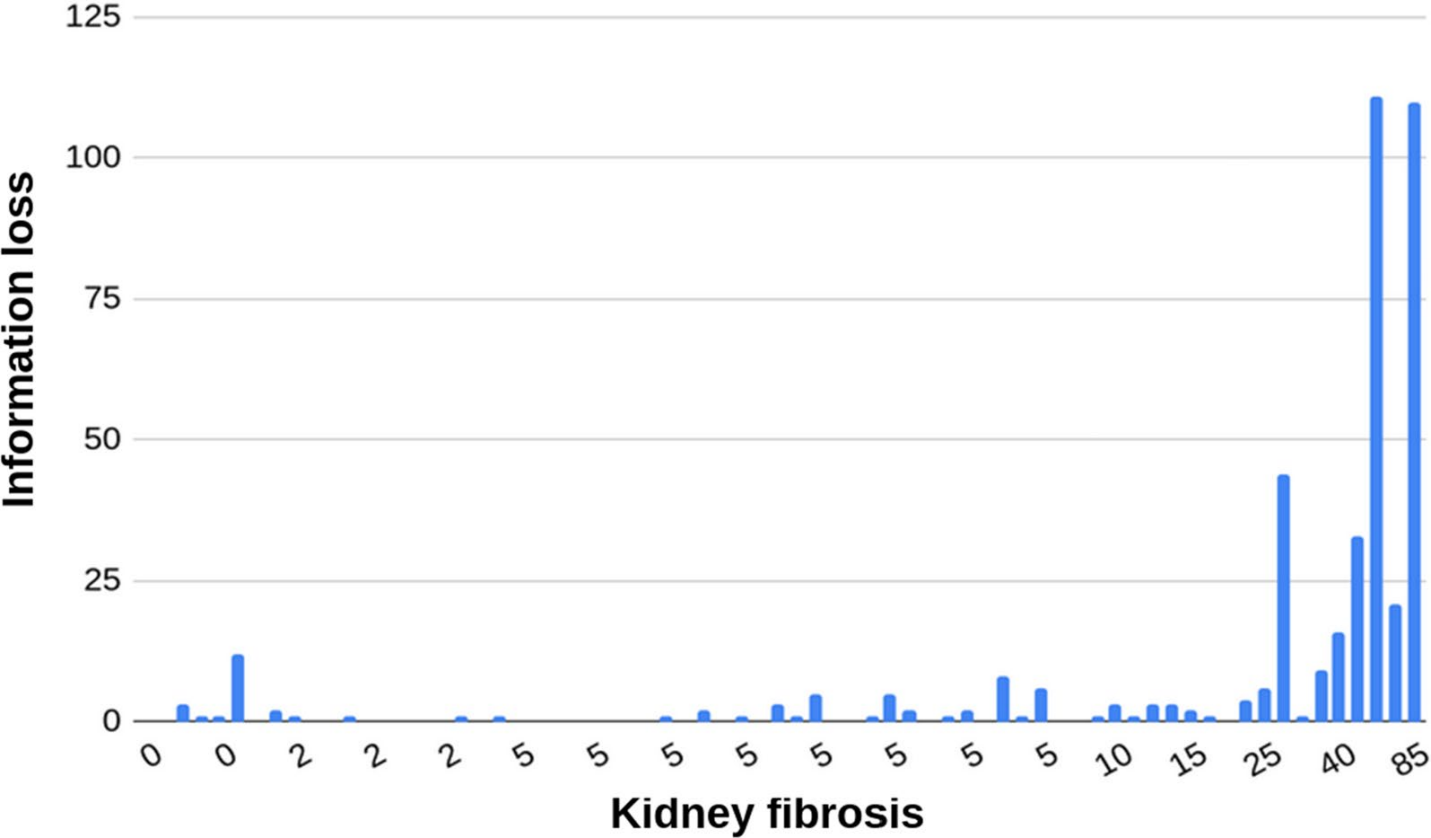
Epigenetic information loss correlates with IF. Epigenetic information loss score for each individual of the GSE50874 dataset (y-axis) along with the corresponding IF score (x-axis)

The discovery that epigenetic information is lost in a specific subset of methylation sites opens up the question of whether a pathological state can be predicted solely based on methylation data, without any other prior knowledge. To answer this question, we implemented a naive information loss score. For each of the 289 unique methylation sites, discovered in the NGDC-CNCB dataset, the kidney median and standard deviation were calculated. Next, for each individual, the number of sites that deviate from the tissue median toward the levels in other tissues, by at least two standard deviations, was calculated (Fig. 6). This score serves as a naive indicator of the loss of epigenetic information specific to kidney tissue. The information loss score showed a *r* = 0.82 correlation with IF (*p* < 10^–18^) and r = − 0.54 with eGFR (*p* < 10^–6^). Thus, the probability of a pathological condition can be inferred solely from the loss of epigenetic information, without prior knowledge, except for the affected tissue.

## Discussion

### Kidney state is captured by specific CpG methylations

Analysis of the kidney methylome revealed that multiple kidney pathologies are captured by distinct epigenetic clocks and surrogates (Fig. 1; Additional file 3: Figure S1), despite the fact that they were developed and tested on different tissues for targeting outcomes different than kidney state. DNAmB2M and DNAmPAI1 had a particularly good correlation with IF in males (*r* = 0.75 and 0.58, respectively). DNAmB2M clock was built based on Beta-2 Microglobulin levels [13]. Increased B2M levels in the blood, and by contrast low levels in the urine, are indicative of glomerular dysfunction [58–60], PAI1 is induced in kidney injury, and has been suggested as a causative agent in CKD [61, 62]. The age B2M and PAI2 surrogates have a low correlation with age (Supp. Tables 2–4). Age-adjusted B2M and PAI2 surrogates significantly correlate to eGFR and IF in males and to IF in females. By contrast, PhenoAge significantly correlates with age, and in females, its strong correlation to eGFR is greatly reduced in the age-adjusted version. (Additional file 1: Tables S2–S4). We interpret these results as B2M and PAI2 surrogates capturing some of the biology of kidney deterioration, mostly independently of aging. By contrast, the correlations generated by PhenoAge are predominantly due to its capturing of the natural aging process. Of note, DNAmB2M and DNAmPAI1 showed relatively low correlation among themselves (r = − 0.30 in males and − 0.14 in females), indicating that an improved estimator can be generated to facilitate more accurate estimation of kidney function across both sexes. Indeed, a global analysis identified multiple methylation sites that, unlike the methylation clocks, show strong correlations for both sexes. A future increase in the number of samples will enable the construction of new epigenetic clocks, capturing kidney function decline with greater precision.

Next, we identified individual CpG sites that correlated with IF and eGFR. As expected, IF and eGFR associated CpGs showed a significant overlap (Additional file 3: Figure S8). On average, IF resulted in stronger correlations than eGFR both for methylation clocks and specific CpGs. This is also expected, as eGFR is an inherently noisy measurement that does not capture the kidney state as accurately as IF [63]. As expected, these results greatly overlapped with [34], which generated and used much of these data with a similar methodology. By contrast, only a small overlap of 21 of 69 CpG sites was observed when compared to blood methylation markers of CKD [21]. We concluded that the physiological state of the kidney is reflected by the methylation levels at multiple sites.

## Tissue-specific methylation patterns are lost with functional decline

Of all sites passing our rigorous threshold for tissue-specificity, 72% have a methylation pattern that correlates with IF (Additional file 1: Table S11). This is likely an underestimation, as the arbitrary cutoff of 10% (*p* < 4** × **10^–5^) is guided by the relatively small dataset used to identify correlated CpGs. The loss of unique epigenetic signature with functional decline appears to be nearly universal in the kidney. Loss of epigenetic information has been suggested to accompany and potentially drive cellular aging [64, 65]. These findings support this hypothesis, and point out that some of the loss may correlate better to functional decline or “biological age” than to chronological age. Moreover, tissue-specific methylation sites appear to be particularly vulnerable to such loss, as most uniquely methylated sites were associated with functional decline, compared to much smaller numbers among all sites. This exceptionally high occurrence leads us to speculate maintenance of these unique sites may serve as a hallmark of functional tissue. Future studies will test if this phenomenon also applies to other tissues, as well as functional decline that does not involve fibrosis.

## Uniquely methylated sites revert to the average

Interestingly, in every single uniquely methylated site tested, the functional decline was accompanied by shift in methylation levels toward the levels common in other tissues. The most plausible explanation for this would be that a default pattern exists in these sites, and a deviation from this default requires active maintenance. Primary DNA sequence is a key driver of local chromatin architecture. The unique methylation patterns could be established and maintained by specific DNA motifs. We tested for DNA motifs that could explain these patterns. While no single motif could explain this unique pattern, we have identified several candidates, enriched in these sites. De novo motif identification discovered not only motifs depleted near unrelated CpG sites, but also motifs present in uniquely methylated sites correlating with IF, but depleted near non-uniquely methylated sites that do correlate to IF.

Two non-exclusive explanations for the loss of epigenetic information are changes to tissue cell composition and changes within the cell populations. For the latter, epithelial to mesenchymal transition (EMT) is a process in which epithelial cells undergo changes that enable them to assume a mesenchymal cell phenotype [66]. EMT has been associated with fibrosis in the kidney [66–69]. Moreover, it has been suggested that changes in DNA methylation causally underlie EMT [70]. Deconvolution analysis (Additional file 3: Figure S3) suggests that changes in tissue cell composition may contribute to this observed change. However, as the methylation profile of each underlying cell population is unknown, the relative contribution of each process remains unknown. Future studies will determine the relative contributions of each of these mechanisms to epigenetic information loss in the kidney.

While this work focused only on kidney-specific methylation patterns, we did observe multiple sites that were shared with one or a few other tissues and were excluded from our lists. It is likely that these findings can be greatly expanded, both by testing sites that show a distinct methylation pattern in other tissues, and by analyzing sites that show a distinct methylation pattern in a subset of tissues.

## Gene expression correlates with changes in methylation

DNA methylation can reflect aspects of local chromatin structure, and may therefore only correlate with gene expression. Alternatively, DNA methylation alters the binding for some TF [33], which in turn affects gene expression. We identified multiple genes associated with CKD-correlated CpG sites that exhibit changes in expression in CKD (Fig. 5; Additional file 3: Table S12). CKD-correlated CpG sites are enriched for JunD TF binding sites. DNA methylation inhibits JunD binding [71, 72], thereby potentially providing a causal mechanism for the regulation of gene expression by methylation at these sites.

The identified kidney-unique undermethylated sites predominantly localize to enhancers. These results are in agreement with the known roles of enhancers in maintaining tissue identity [73, 74], and the changes in enhancer regions of core pro-fibrotic genes observed in kidney fibrosis development [22]. These results suggest that the loss of open chromatin on these key enhancers may be part of the loss of kidney-unique epigenetic signature and cell identity.

## Materials and methods

Demographics of the datasets are available as (Additional file 3: Tables S14–S15).

## Epigenetics clocks and CpG site correlations

Epigenetic estimators were calculated using the DNA Methylation Age Calculator [14] and the clock foundation web server (dnamage.clockfoundation.org) for the 85 Illumina HumanMethylation450 BeadChip array samples for which full pathological data was available (GEO GSE50874). Samples that generated the warnings “meanMethBySample < 0.25” were removed and the data was re-analyzed (Additional file 1: Table S1), resulting in a very mild increase in most correlations. Next, samples separated into males and females and analyzed again (Additional file 1: Table S3–S4). All CpG sites were scored by Pearson correlation (*r*) to eGFR and IF. For each *r*, *t* = *r*√(n-2) / √(1-r^2^) was calculated, along with the corresponding p-value. Age and sex were were excluded as confounders by testing for association with eGFR and IF both the GSE50874 and the NGDC-CNCB dataset. Partial correlations were calculated using the pg.partial_corr function from the pingouin python package.

## Identification of uniquely methylated sites

The National Genomics Data Center, China National Center for Bioinformation (NGDC-CNCB; [35] dataset contains tissue-level methylation data across multiple partially annotated individuals. It was used to infer tissue-specific methylation levels. Uniquely methylated sites were defined as such that the average methylation level in kidney is lower by at least 0.2 than the 5% quantile level of all other samples in the NGDC-CNCB dataset, or higher by at least 0.2 than the 95% quantile level of all other samples. This distinguished methylation sites that out of the 28 tissues in the dataset, have a unique signature in the kidney, but did not exclude samples where a very small minority of samples leaks throughout the entire range. Next, CpG sites with less than 2000 values were removed. These sites were identified using custom R and Python scripts.

## Motif Enrichment

Motif enrichment analysis for CKD-correlated CpG sites was done using MonaLisa [75], using 200 bp of sequence from each side of each CpG site. As a control, CpG sites showing no correlation were inserted between the positively and negatively correlated sites. De Novo identification of motifs near uniquely methylated sites was done using Homer [76] on 500 bp of sequence from each side of each CpG site. These were compared to randomly selected CpG sites, or IF correlated randomly selected CpG sites.

## DHS and chromatin state analysis

DHS and chromatin state analysis was performed using the eFORGE 2.0 web server (https://eforge.altiusinstitute.org/), using the “Consolidated Roadmap Epigenomics—DHS” and the “Consolidated Roadmap Epigenomics—Chromatin—All 15 state marks,” respectively. Default setting of 1 kb window, 1000 background repetition, 0.01 strict and 0.05 marginal were kept.

## Deconvolution analysis

Deconvolution analysis was performed using the EpiSCORE web server (www.biosino.org/EpiDISH/) with “kidney” reference.

## Gene expression analysis

Genes associated with all uniquely methylated sites CpG sites were extracted from the HumanMethylation450 v1.2 Manifest File (Illumina, USA). Gene expression levels in CKD and control kidney biopsies from the Nakagawa CKD Kidney dataset [45] were extracted using NephroSeq.

### Supplementary Information

**Additional file 1**. Supplementary Tables 1–15.

**Additional file 2**. Supplementary Figure 7.

**Additional file 3**. Supplementary Figures 1–8.

## Data Availability

Data derived from public domain resources. New analysis data are available in the supplementary material. Code is available at https://github.com/TheBarLab/Kidney/.
