# Supplementary material for: Kidney-specific methylation patterns correlate with kidney function and are lost upon kidney disease progression
Source: Clin Epigenetics. 2024 Feb 12;16:27. doi: 10.1186/s13148-024-01642-w (PMC10863297; doi:10.1186/s13148-024-01642-w)
Supplement: Supplementary file 3 — Additional file 3. Supplementary Figures 1–8. [file 13148_2024_1642_MOESM3_ESM.docx]

**Supplementary figures**


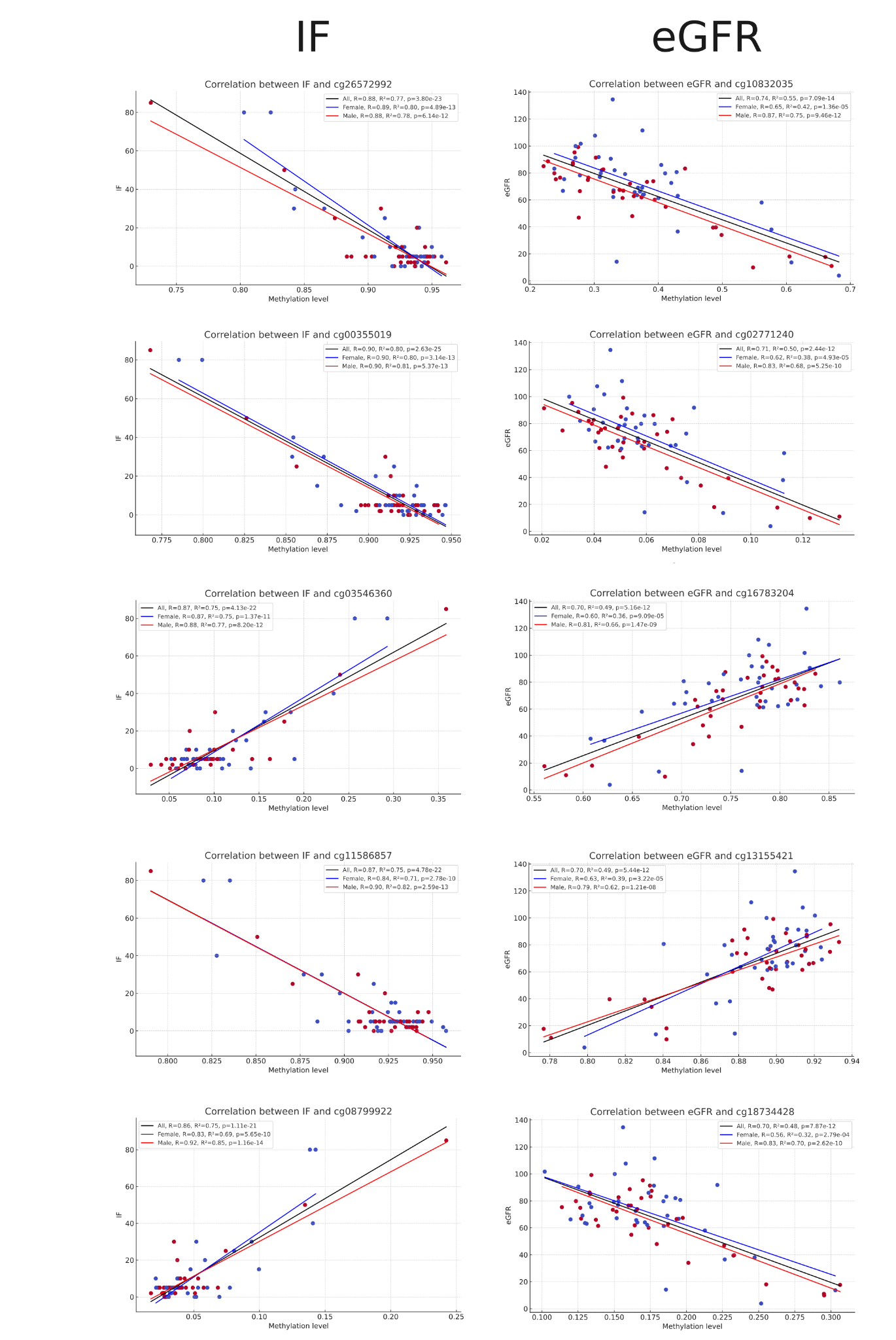


***Supplementary figure 1: Regression analysis for top 5 sites (Supp. Table 4-5) for IF (left) and eGFR (right).*** *Methylation level is plotted on the x axis, and IF or eGFR on the y axis. Males - red; Females - blue. Regression line with all samples - black.*


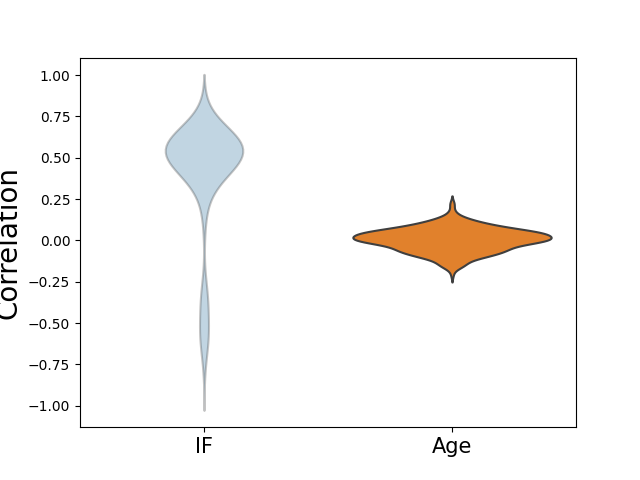


***Supplementary figure 2: Correlation distribution between IF and methylation levels (left violin plot) and between Age and methylation levels in kidney unique sites (right violin plot).***


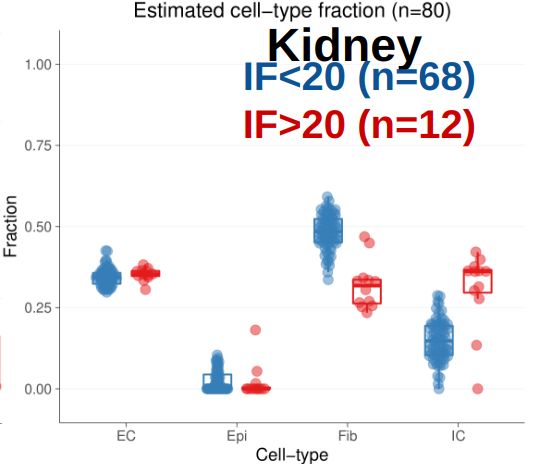


***Supplementary figure 3: EpiSCORE estimation of cell composition in kidney samples with IF above or below 20.***


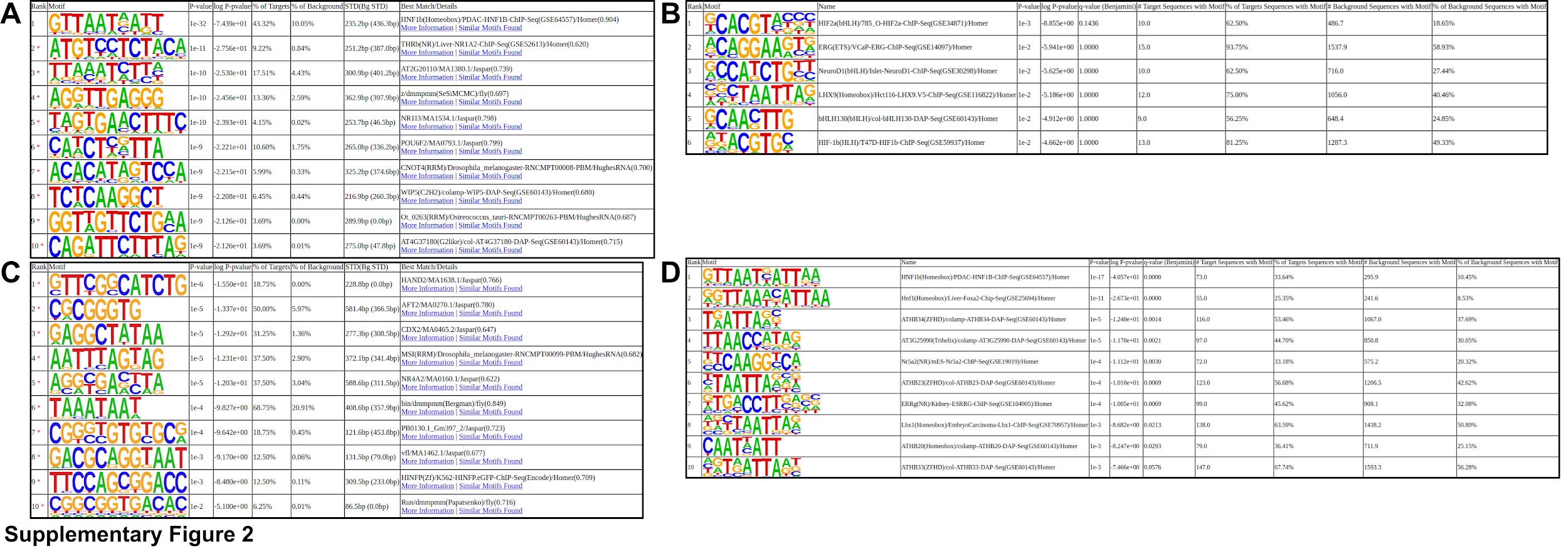


***Supplementary figure 4: Motif enrichment in uniquely methylated sites vs. random sites.*** *De novo (****A****) and known (****B****) motif enrichment in sites undermethylated in the kidney. De novo (****C****) and known (****D****) motif enrichment in sites overmethylated in the kidney. Up to top 10 results are displayed. * - possible false positive.*


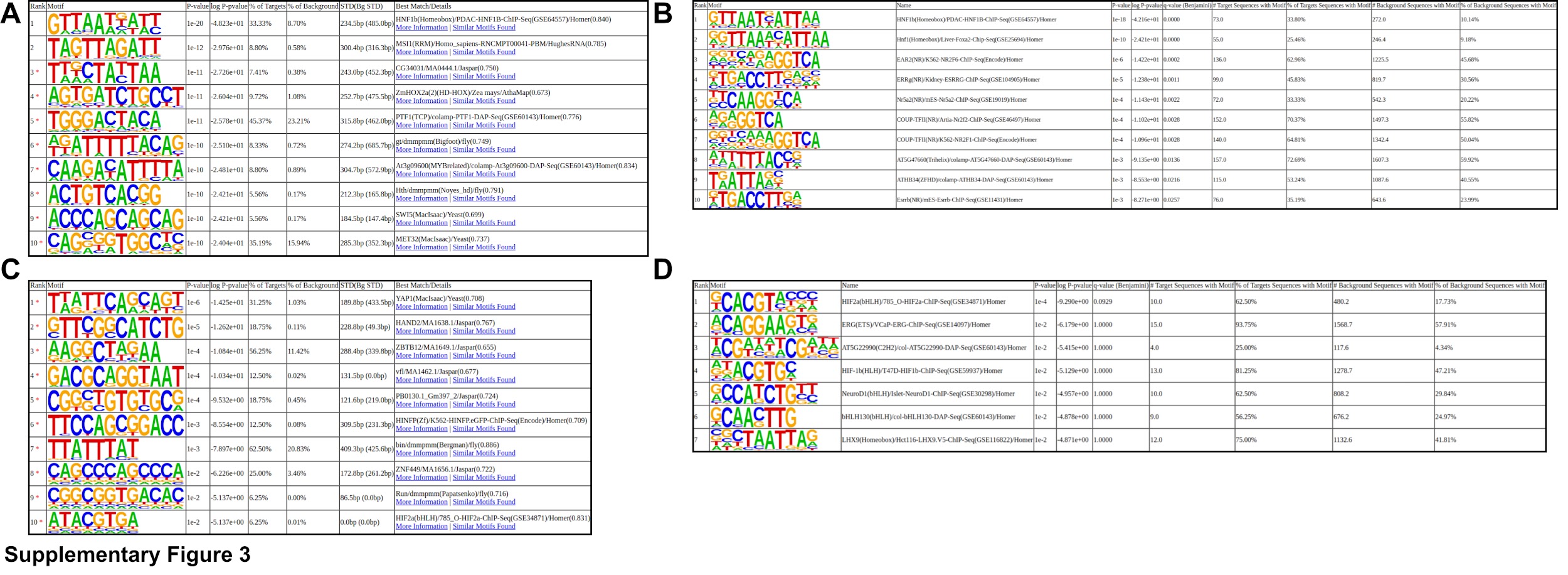


***Supplementary figure 5: Motif enrichment in uniquely methylated sites vs. IF-correlated sites.*** *De novo (****A****) and known (****B****) motif enrichment in sites undermethylated in the kidney. De novo (****C****) and known (****D****) motif enrichment in sites overmethylated in the kidney. Up to top 10 results are displayed. * - possible false positive.*


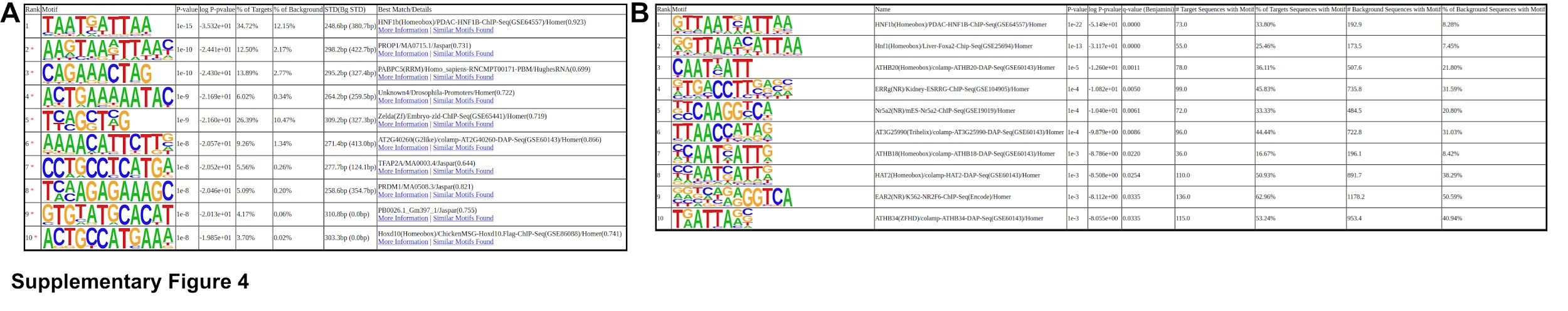


***Supplementary figure 6: Motif enrichment in sites uniquely methylated in kidney vs. other tissues.*** *De novo (****A****) and known (****B****) motif enrichment in sites undermethylated in the kidney. Up to top 10 results are displayed. * - possible false positive.*


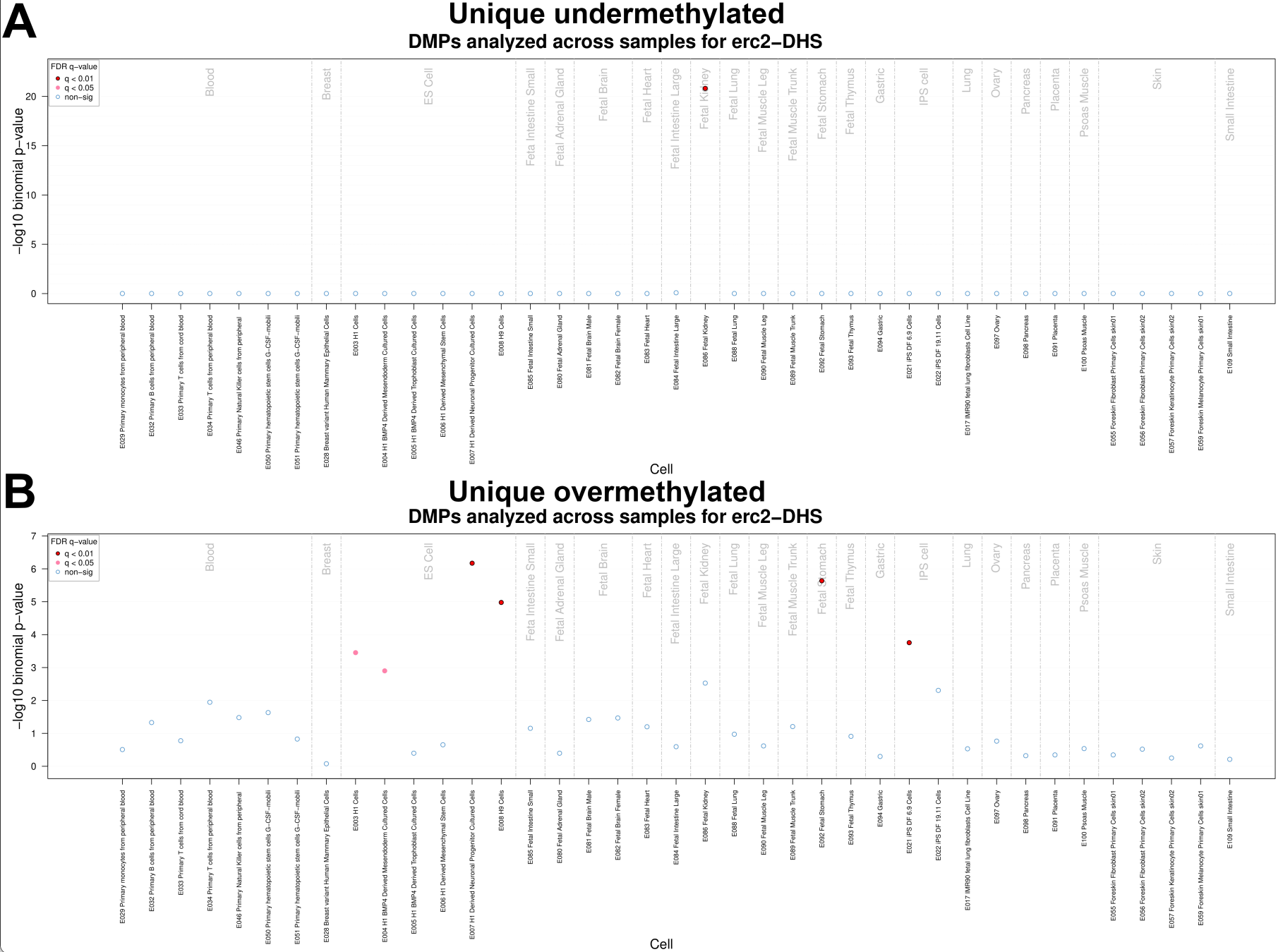


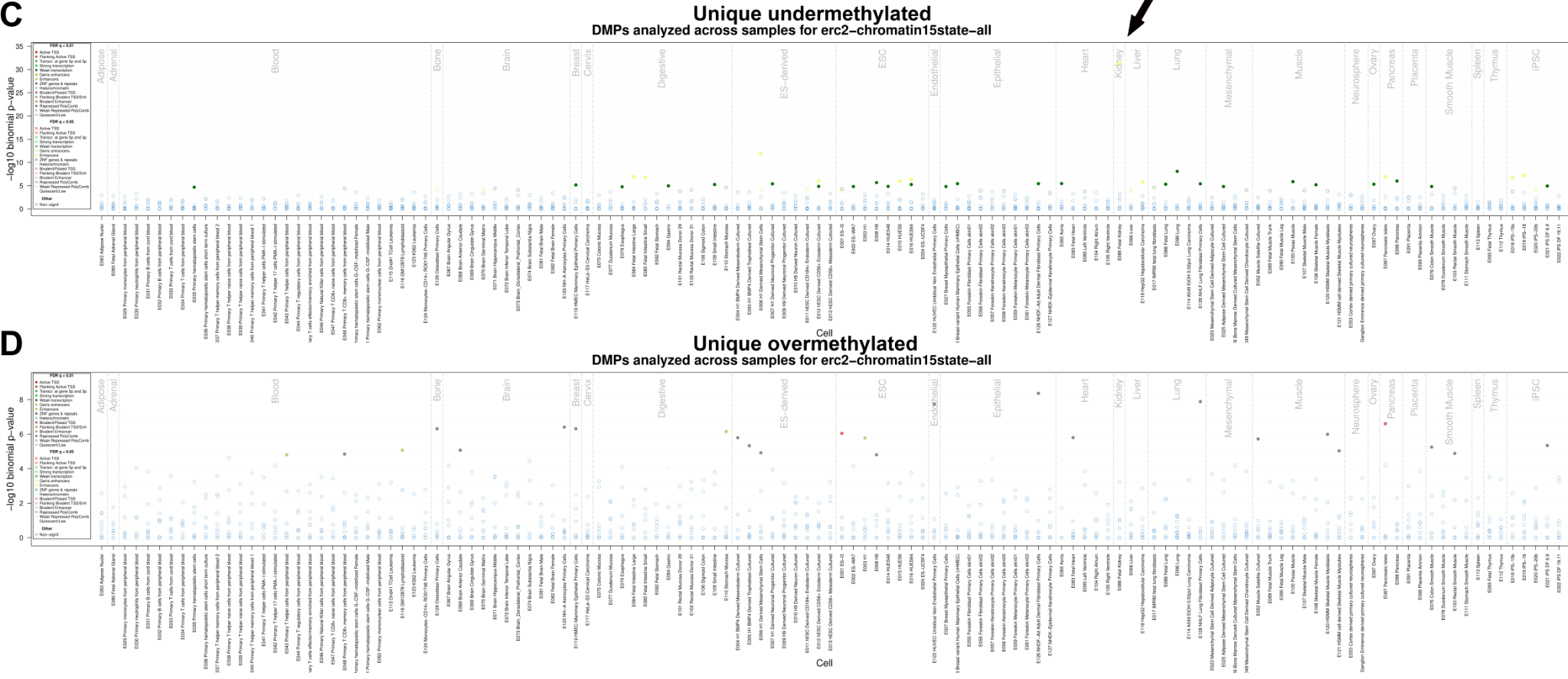


***Supplementary figure 7: Genomic accessibility and function of kidney-unique methylation sites.*** *Tissue-specific DMP analysis of kidney-unique undermethylated (****A****) and overmethylated (****B****) sites. Tissue-specific chromatin state analysis of kidney-unique undermethylated (****C****) and overmethylated (****D****) sites.*


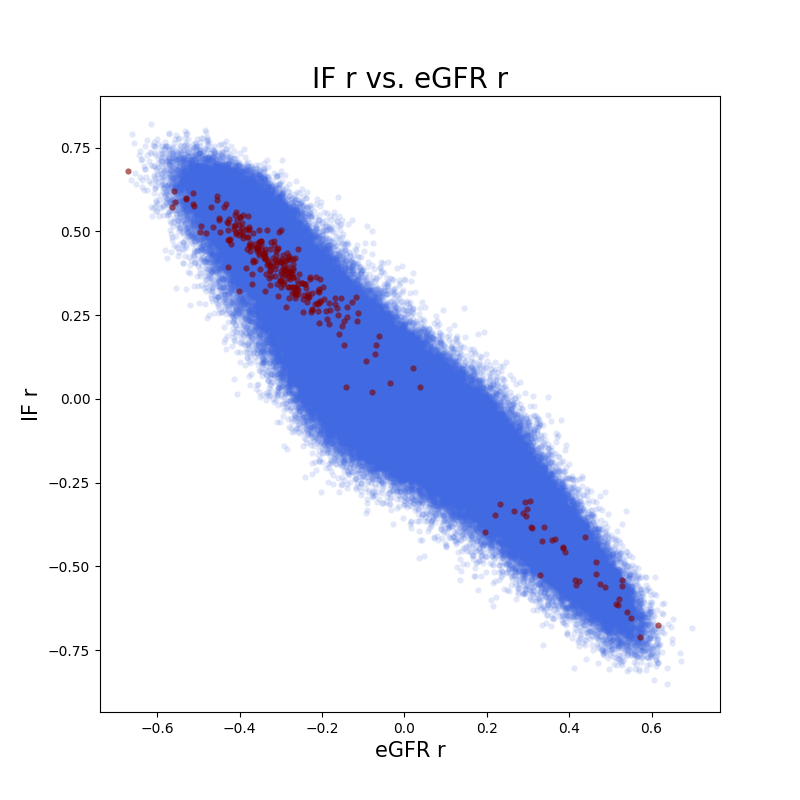


***Supplementary figure 8: Correlation of IF-methylation levels Vs. correlation of eGFR-methylation levels.*** *Unique sites (red dots) and non-unique sites (blue dots). Note the small amount of red dots near zero.*
